# Analysis of the gut microbiome in dogs and cats

**DOI:** 10.1111/vcp.13031

**Published:** 2021-09-12

**Authors:** Jan S. Suchodolski

**Affiliations:** ^1^ Gastrointestinal Laboratory Department of Small Animal Clinical Sciences College of Veterinary Medicine and Biomedical Sciences Texas A&M University College Station TX USA

**Keywords:** cats, *Clostridium hiranonis*, dogs, Dysbiosis Index, fecal microbiota transplantation, metagenomics, microbiome

## Abstract

The gut microbiome is an important immune and metabolic organ. Intestinal bacteria produce various metabolites that influence the health of the intestine and other organ systems, including kidney, brain, and heart. Changes in the microbiome in diseased states are termed dysbiosis. The concept of dysbiosis is constantly evolving and includes changes in microbiome diversity and/or structure and functional changes (eg, altered production of bacterial metabolites). Molecular tools are now the standard for microbiome analysis. Sequencing of microbial genes provides information about the bacteria present and their functional potential but lacks standardization and analytical validation of methods and consistency in the reporting of results. This makes it difficult to compare results across studies or for individual clinical patients. The Dysbiosis Index (DI) is a validated quantitative PCR assay for canine fecal samples that measures the abundance of seven important bacterial taxa and summarizes the results as one single number. Reference intervals are established for dogs, and the DI can be used to assess the microbiome in clinical patients over time and in response to therapy (eg, fecal microbiota transplantation). In situ hybridization or immunohistochemistry allows the identification of mucosa‐adherent and intracellular bacteria in animals with intestinal disease, especially granulomatous colitis. Future directions include the measurement of bacterial metabolites in feces or serum as markers for the appropriate function of the microbiome. This article summarizes different approaches to the analysis of gut microbiota and how they might be applicable to research studies and clinical practice in dogs and cats.

## 1 IMPORTANCE OF THE INTESTINAL MICROBIOME IN HEALTH AND DISEASE

The gastrointestinal (GI) tract harbors a complex ecosystem consisting of various microbes such as bacteria, viruses, fungi, and protozoa. This system is termed microbiota when referring to taxonomy (“who is there”) and microbiome when referring to their gene content and function (“what are they doing”). Bacteria constitute by far the largest component of these intestinal microorganisms, with >98% of metagenomic sequencing reads from fecal samples being assigned to bacteria in dogs and cats.^1^, ^2^ Fungal organisms have been identified as a normal component of the microbiota in the small and large intestine, but their contributions to health and disease remain unknown.^3^, ^4^

The gut microbiome consists mostly of strict or facultative anaerobic bacteria, especially in the highly populated large intestine. The predominant phyla in dogs and cats are Firmicutes, Fusobacteria, and Bacteroidetes.^5^, ^6^ Intestinal bacteria either produce or convert dietary molecules or drugs into bacteria‐derived metabolites, and the gut microbiome is considered an important metabolic organ. A balanced gut microbiome exerts a beneficial influence on host health by modulation of the immune system, defense against intestinal pathogens, and provision of vitamins and nutrients. For example, dietary carbohydrates are fermented by bacteria into short‐chain fatty acids (SCFA), which provide energy for epithelial cells, regulate intestinal motility, and possess anti‐inflammatory properties.^7^ Many other bacterially derived metabolites have beneficial properties. Examples are indole,^8^ a bacterial degradation product of the dietary amino acid tryptophan, and secondary bile acids that are converted by intestinal bacteria from primary bile acids secreted by the liver.^9^

These microbial effects reach beyond the GI tract. Studies in dogs and cats show that changes in the intestinal microbiome and/or function are not only present in GI disease,^10^, ^11^ but are also associated with disorders in other organ systems such as chronic kidney disease (CKD),^12^ heart disease,^13^, ^14^, ^15^ neurologic disorders,^16^ diabetes mellitus,^17^ and obesity.^18^ While the exact underlying mechanisms still need to be explored in many of these disorders, some microbial pathways are now well‐recognized contributing to health and disease (Table 1), and some of these can be directly assessed using different methods. A better understanding of the gut microbiota and their function will lead to advances in new diagnostic and therapeutic options. This article summarizes different approaches to the analysis of gut microbiota and how they could be applicable to research studies as well as clinical practice.

**TABLE 1:** Contribution of intestinal bacteria to metabolic pathways that influence health and disease

Source	Bacteria involved	Microbial metabolite(s)	Effects on host	
			Beneficial when in normal concentrations	Potentially deleterious when in abnormal concentrations
Dietary carbohydrates	Various (eg, *Faecalibacterium, Bifidobacterium*)	Fermentation to short‐chain fatty acids	Anti‐inflammatory properties	Abnormal SCFA ratio can activate virulence factors of enteropathogens (eg, Salmonella invasion genes, *Escherichia coli* motility)
			Improve barrier function	
			Regulate intestinal motility	
			Provide systemic and local energy	
Primary bile acids from liver	Mostly *Clostridium hiranonis* in dogs and cats	Transformation to secondary bile acids (BA)	Anti‐inflammatory	Increased primary BA can lead to secretory diarrhea
			Secondary BA are a major regulator of normal microbiome, also inhibit growth of *C difficile, C perfringens*, *E coli*	
Tryptophan from diet	Various	Indole metabolites	Anti‐inflammatory, maintain intestinal barrier function	In increased concentrations cytotoxic, putrefactive indoxyl sulfate acts as uremic toxin
Dietary carnitine and choline	Various (eg, *E coli*)	Trimethylamine N‐oxide (TMAO)	n/a	Altered cholesterol metabolism associated with heart disease

Abbreviation: n/a, not applicable.

## 2 ASSESSMENT OF THE INTESTINAL MICROBIOME—GENERAL CONSIDERATIONS

It is important to emphasize that intestinal bacteria constitute just one part of an intricate relationship that exists between the intestinal epithelial cells, the intestinal mucus layer, the host immune system, and the luminal environment. The composition of the microbiota is influenced to some degree by diet, drugs such as antibiotics and chemotherapeutics, inflammation in the gut, structural changes in the intestine, and others.^19^, ^20^, ^21^ Some of these factors have been recently reviewed in detail elsewhere.^22^ Therefore, studies should aim to evaluate these mechanisms using complementary approaches (taxonomic and functional) to understand how specific bacteria are modulated by the microenvironment within the gut and under which situations they contribute to health and disease.

There are differences in bacterial populations between the stomach, and the small and large intestine, mostly due to differences in intestinal physiology (difference in oxygen levels, pH, antimicrobial compounds, and intestinal motility). The canine stomach harbors only a few types of bacteria that can survive the acidic environment, predominantly *Helicobacter* spp. and, to a smaller degree, lactic acid‐type bacteria.^23^ The small intestine harbors a mix of aerobic and anaerobic bacteria.^24^, ^25^ The large intestine is highly populated with mostly anaerobic bacteria.^5^, ^6^

**FIGURE 1:**
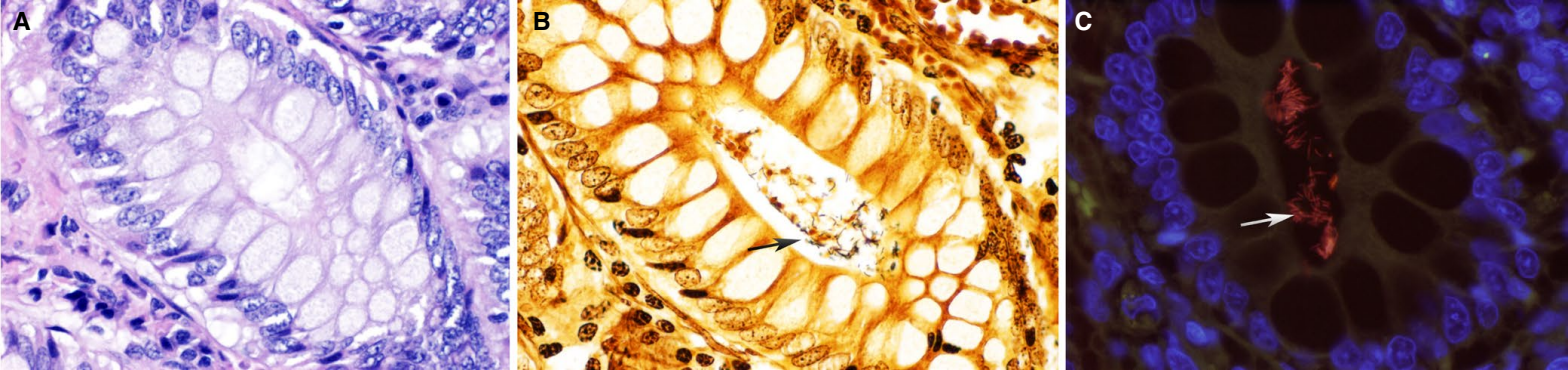
Photomicrograph of the colonic mucosa of a healthy dog. The bacteria within the crypts of healthy dogs are inconspicuous on routine hematoxylin and eosin stain (A). The Steiner silver stain (B) highlights abundant bacteria (arrow) within the crypts. Fluorescence in situ hybridization with EUB338 probe targeting all bacteria in the crypts. Labeled bacteria appear red (arrow). The autofluorescence of the intestinal mucosa appears green. DAPI (4′,6‐diamidino‐2‐phenylindole)‐stained nuclei of colonic mucosa appear blue. ×60 objective. Courtesy of Dr Paula Giaretta, DACVP, Universidade Federal de Minas Gerais, Brazil^27^

Most studies have evaluated the fecal microbiome, as this is the most accessible sample type in clinical settings. Yet, the analysis of fecal samples does not provide complete information about the potential presence of mucosa‐adherent or entero‐invasive bacteria or the composition and the quantity of the small intestinal microbiota. There are differences in luminal vs mucosa‐adherent bacterial populations, and for some disorders, the assessment of mucosa‐adherent bacteria by fluorescence in‐situ hybridization (FISH) might be useful.^10^, ^21^, ^26^ A recent study used FISH (Figure 1) to describe bacteria (*Helicobacter* spp.) deep in the colonic crypts of healthy dogs, and bacteria in these locations could have important immunological properties for health and disease as compared with luminal bacteria.^27^ The small intestinal microbiota, even if of normal composition, can contribute to clinical signs when there is an abnormal or increased amount of food or drug substrate in the intestinal lumen. This can be due to feeding diets with poor digestibility, inflammatory diseases that damage the transporters in the epithelial brush border,^28^, ^29^ and a lack of digestive enzymes in patients with exocrine pancreatic insufficiency (EPI).^30^ Therefore, abnormal microbial conversion of luminal substrates by normal microbiota can be pathologic, not just changes in bacterial populations. While some of the microbiome changes that likely originate in the small intestine can be detected in fecal samples, as reported for dogs receiving omeprazole,^23^, ^31^ dogs with EPI,^32^ and dogs with chronic enteropathies (CE),^33^, ^34^ the above limitations should nevertheless be considered when analyzing fecal samples only.

The intestinal microbiota is highly diverse in phylogeny, and despite advances in molecular methods used to characterize this ecosystem, it is still difficult to describe all bacteria present. Frequently, methods used based on 16S ribosomal RNA (rRNA) gene sequencing do not have sufficient resolution to identify all bacteria, especially at the species and strain level.^35^, ^36^ Furthermore, depending on the intestinal microenvironment (eg, pH, nutrients, specific metabolites), similar bacterial species can express different genes and, therefore, have different metabolic functions. The expression of bacterial virulence factors may depend on the concentrations of metabolites in the intestinal lumen. For example, the presence of the stress hormone norepinephrine in the gut modifies *Salmonella* genes to induce enteritis.^37^ Changes in the ratios of the SCFAs butyrate, propionate, and acetate influence the expression of virulence factors of *Salmonella enterica*.^38^ *Escherichia coli* exhibits different growth rates and motility in the ileum vs colon, and this is dependent on differences in the SCFA ratio between these two intestinal sites.^39^ Bile acid metabolism is another important bacterial‐derived metabolic pathway that, when disrupted, will lead to overgrowth of potential enteropathogens. A disrupted microbiota can lead to a decreased abundance of intestinal bacteria that are able to convert primary to secondary bile acids, which in turn allows the overgrowth of *Clostridioides difficile* in the colons of people.^40^

These examples illustrate that for clinical research purposes, the analysis of gut microbiota and their functions should encompass complementary approaches, such as the phylogenetic assessment of bacteria present (eg, 16S rRNA gene sequencing, PCR for bacteria), their functional genes (DNA shotgun sequencing, PCR for functional genes), and microbial‐derived metabolites. Furthermore, detailed patient information should be collected, such as medication and diet history, a final diagnosis, and information about long‐term clinical outcomes. Samples should be evaluated over the disease course, and all information should be correlated with microbiome data to better understand the contributions of these pathways to disease. One important example of such a comprehensive approach is the identification of *Clostridium hiranonis* as the main converter of primary to secondary bile acids in dogs, and due to this function, a decreased abundance of this bacterium leads to shifts in the intestinal microbiota.^15^, ^41^, ^42^, ^43^, ^44^, ^45^ A disruption of the microbiome and decrease in *C* *hiranonis* can be induced by broad‐spectrum antibiotics,^42^, ^43^ and is often present in chronic inflammatory enteropathies.^11^, ^29^, ^41^ Several recent studies have shown that a decrease in the abundance of this bacterium is highly associated with dysbiosis (see below under dysbiosis index).^29^, ^43^ Therapeutic modulation with diet or fecal microbiota transplantation can lead to the normalization of *C hiranonis* abundance, which is associated with normalization of the microbiota.^41^, ^42^, ^44^ Another important bacterium is *Faecalibacterium prausnitzii*, which produces short‐chain acid and anti‐inflammatory peptides and is often decreased in canine and feline intestinal diseases.^29^, ^43^, ^46^

## 3 BACTERIAL CULTURE

As mentioned above, the majority of intestinal bacteria, especially in the large intestine, are strict anaerobes, and most require special growth media. Some specialized research laboratories are able to cultivate these microbes through a combination of molecular tools to identify bacteria in a sample and then optimize culture conditions for their growth.^47^

Traditional bacterial culture, however, as performed in veterinary diagnostic laboratories, vastly underestimates the number of intestinal bacteria because only standard bacterial media and/or limited anaerobic methods are used. Only a small percentage of bacterial species can be isolated from the feces of clinical patients, and these have been reported by commercial diagnostic laboratories. Unfortunately, because these bacteria are isolated from clinical patients; clinicians often erroneously consider them as pathogens (eg, *E coli*, *C perfringens*).^48^ In a recent study, three aliquots of fecal samples from healthy dogs and dogs with chronic diarrhea were submitted in a blinded fashion to three veterinary reference laboratories for the evaluation of dysbiosis or the culture of pathogenic bacteria. The authors ordered a so called “fecal bacterial culture profile”.^48^ Across all samples, bacterial culture results from all three laboratories did not reveal significant differences in microbiota between healthy dogs and dogs with chronic diarrhea. Interestingly, the laboratories reported dysbiosis more frequently in healthy dogs, and there was no agreement in the reported culture results between the three laboratories.^48^ Hemolytic *E coli* were more frequently isolated from healthy dogs than dogs with diarrhea. This was in contrast with the molecular‐based Dysbiosis Index (see below), which was significantly higher, indicating dysbiosis in dogs with chronic diarrhea.^48^

These results are not surprising due to the lack of bacterial culture standardization between laboratories, unknown criteria for how a microbiota dysbiosis has been defined by each laboratory, and the fact that most bacteria in the gut are anaerobes and therefore remain undetected. These anaerobic bacteria, which provide various metabolic benefits to the host (Table 1), are typically reduced in acute and chronic intestinal disease.^49^, ^50^ It is likely that this reduction in beneficial bacteria and, therefore, microbiome function is clinically more important than an overgrowth of individual facultative cultivable bacterial species (eg, *C perfringens*).^51^, ^52^ Examples are reductions in SCFA and anti‐inflammatory peptides producing bacteria (eg, *Faecalibacterium*) and bile acid converting bacteria such as *C hiranonis,* both of them not cultivable with standard aerobic techniques.^49^, ^53^ There is a need to educate clinicians about the complexity and functionality of the intestinal microbiome, which cannot be assessed by culture. However, bacterial culture remains important to test for antibiotic susceptibility of those few cultivable organisms that are known to be associated with intestinal infections (eg, *Salmonella* spp.). Bacterial culture and susceptibility profiling of invasive *E coli* from colonic biopsies of dogs with granulomatous colitis is also recommended, as recent data has shown that these entero‐invasive organisms are often resistant to many of the previously recommended antimicrobials (ie, fluoroquinolones).^54^

## 4 NEXT‐GENERATION SEQUENCING

Next‐generation sequencing (NGS) includes sequencing of 16S rRNA genes, DNA shotgun sequencing (metagenomics), and metatranscriptomics (Table 2). The latter approach attempts to assess the gene expression of intestinal microbes but is currently a rarely used method due to the expense and the complexity of analysis.^55^ Almost all studies assessing the intestinal microbiota in companion animals used sequencing of 16S rRNA genes, and only a few studies performed deep DNA shotgun sequencing.^1^, ^2^, ^56^, ^57^

**TABLE 2:** Commonly used methods for characterization of the intestinal microbiota

Method	Purpose	Description	Advantages	Disadvantages
Fluorescence in situ hybridization (FISH)	identification, quantification, visualization of bacterial cells in tissue	fluorescent dye‐labeled oligonucleotide probes are hybridized to ribosomal RNA sequence in bacterial cells	useful method for quantifying bacteria, allows localization of bacteria in tissue	labor intense, FISH probes need to be developed for each group of interest
Quantitative real‐time PCR	quantification of bacterial taxa	target organisms are quantified using fluorescent dye‐labeled primers and/or probes	rapid, reproducible, inexpensive, quantitative, RIs can be established	primer and probes need to be designed for each group of interest
16S rRNA sequencing	identification of bacteria in a sample, measures relative abundance	bacteria are amplified using universal primers targeting the 16S rRNA gene, PCR amplicons are separated and sequenced using a high‐throughput sequencer	high throughput, relative inexpensive, allows identification of bacteria, semi‐quantitative, allows to describe changes within a community	requires advanced bioinformatics, changes in taxonomic databases and bioinformatics pipelines make comparing results difficult across studies, does not allow to detect changes in total abundance of bacteria
Metagenomics (shotgun sequencing of genomic DNA)	identification of microbial genes present in sample	genomic DNA is fragmented and then randomly sequenced (without PCR amplification) on a high‐throughput sequencer	provides not only phylogenetic information but also what functional genes are present in sample	expensive, requires advanced bioinformatics, does not allow to detect changes in the total abundance of bacteria

### 4.1 DNA shotgun sequencing (metagenomics)

Deep DNA shotgun sequencing, or metagenomics, aims to sequence extracted DNA in a sample without prior amplification by PCR.^58^ Using this approach, various genes are identified, allowing assessments of taxonomy and functional gene categories (“what they can do”) in the microbiome of samples (eg, synthesis of amino acids, vitamins, carbohydrate). Metagenomics has better resolution at the species and strain level compared with 16S rRNA gene sequencing, as multiple genes from one organism can be sequenced and overlapping reads can be assembled to a draft genome. This approach also identifies other members of the intestinal community, such as archaea, fungi, and DNA viruses. Metagenomic analysis of fecal samples from dogs revealed that bacteria makeup approx. 98% of all sequencing reads, archaea 1%, and fungi and DNA viruses (mostly bacteriophages) the remaining 1%.^1^ Similar proportions were observed in the fecal samples of cats.^56^ Metagenomics also provides more detailed information about the presence of virulence genes and antimicrobial resistance genes.^2^, ^57^ Therefore, metagenomics would be the preferred method for analysis of the gut microbiota in research studies. Unfortunately, this approach is rarely used in veterinary medicine, mostly due to the much higher costs compared with 16S rRNA gene sequencing, as a very deep sequencing coverage is required to detect important functional genes that make up only a small percentage of obtained sequences. Furthermore, advanced bioinformatics is required to assemble all data using different bioinformatics pipelines and databases.^59^, ^60^, ^61^ Therefore, deep metagenomics is often cost‐prohibitive for studies involving a large number of animals or time points. A novel approach, called shallow shotgun metagenomics, could be a potential alternative and uses lower sequence coverage (ie, fewer sequencing reads).^62^ Because of lower coverage, the costs are much less than for deep metagenomics and only slightly higher than those for 16S rRNA gene sequencing. This method provides more accurate phylogenetic data (who is there) about a species level than 16S rRNA gene sequencing, in addition to the functional gene content. Shallow shotgun metagenomics has been used to assess the human microbiome^62^ and will likely be more commonly used in companion animals in the future. However, it is yet unknown whether this shallow sequencing approach will provide similar information as deep sequencing for rare members of the community such as fungi, viruses, and archaea, and rare functional genes.

The viral community is also an important part of the intestinal ecosystem. However, very little additional information is available about the viriome beyond those viruses that have been described with targeted approaches such as parvoviruses and coronaviruses. Describing the entire viriome is challenging, as it consists of DNA and RNA viruses, and they are phylogenetically inhomogeneous. Therefore, a deep sequencing approach that combines the analysis of DNA and RNA is required. Only a few studies have been performed in veterinary medicine, and these have identified various bacteriophages and novel viral families and genera in healthy and diarrheic dogs.^63^, ^64^ Because of the cost associated with the analysis, only a small number of dogs have been evaluated; and therefore, no strong conclusions can be made about the role of these novel viruses in intestinal disease. Nevertheless, these approaches allow identification of new members of the intestinal community, which can be followed in a larger population of animals using more targeted approaches (eg, PCR).

### 4.2 16S rRNA gene sequencing

The most frequently used sequencing technique for the assessment of intestinal bacteria in dogs and cats uses 16S rRNA gene sequencing. Briefly, DNA is extracted from intestinal samples, such as biopsies, luminal content, or fecal samples. The 16S rRNA gene consists of several variable regions, each flanked by conserved regions. Bacterial primers are used that amplify these conserved regions and, thus, the variable region in‐between. Because conserved regions are targeted, in theory, DNA from known and unknown bacteria present in the sample can be amplified, and then variable regions can be sequenced. This process requires specific library preparations for different sequencing platforms (eg, Illumina MiSeq, Ion Torrent PGM).^65^ The obtained raw sequences are then processed through a bioinformatics pipeline, such as QIIME 2 or Mothur,^66^, ^67^ to eliminate sequences of insufficient quality and erroneous reads, remove chimeric sequences, and compare the final sequences against public databases.^60^, ^68^, ^69^ Statistical analysis is performed according to the study design. The basic analysis compares alpha diversity (the richness and diversity of a sample, ie, how many taxa has one sample), beta diversity (how similar is one sample to another based on taxa present), and the individual bacterial taxa on all phylogenetic levels between groups and/or treatments.^59^ There are several easy to use web‐based interfaces that allow statistical analysis and visualization of microbiome data, such as Calypso (http://cgenome.net/wiki/index.php/Calypso)^70^ or MicrobiomeAnalyst (https://github.com/xia‐lab/MicrobiomeAnalystR).^71^

Sequencing of 16S rRNA genes is currently the standard approach in microbiome studies and provides useful in‐depth information about the microbial composition and how individual bacterial groups or the entire community differ between healthy vs diseased animals or respond to dietary or therapeutic interventions. This method is useful to detect overall differences in microbiota composition but is not reliable to detect which exact bacterial species are causing those changes. Furthermore, it is important to realize that there is considerable variation in the methods used between studies. Furthermore, the sequencing platforms, bioinformatics pipelines, and available phylogenetic databases are in constant evolution over time, even when performed in the same laboratory. Therefore, like any biomarker testing, comparing data within the same study and/or methods is useful and appropriate, but results should be carefully interpreted across studies performed using different methods and across different periods.

**FIGURE 2:**
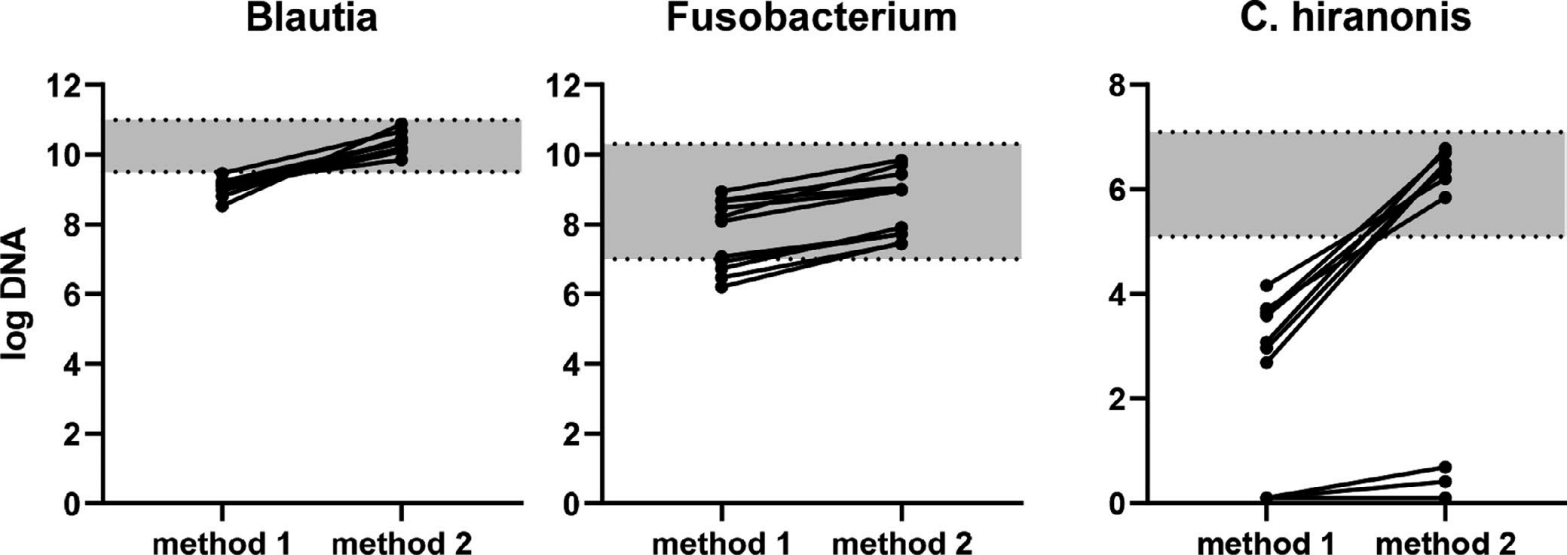
Effect of the DNA extraction method on the abundance of fecal bacteria. Two different DNA extraction methods were compared for canine fecal samples, and the bacterial taxa were measured using identical quantitative PCR (qPCR) assays.^49^ Method 1 uses chemical lysis, whereas method 2^49^ employs bead beating in addition to chemical lysis. Grey areas indicate the RIs for the targeted bacteria. Differences in methods will affect the measured the abundance in 16S rRNA gene sequencing and qPCR data. It is possible to establish RIs for specific taxa, but assays need to be analytically validated and performed with proper quality control to reproducibly assess the microbiota across studies and in clinical settings

In sequencing studies, the abundance of bacterial taxa are expressed as relative proportions of the total bacterial community and then statistically compared between treatment groups. There are several factors that will affect the reported relative proportions. The method of DNA extraction (eg, bead‐beating vs non‐bead‐beating, addition of lysozyme, RNAse treatment) will affect the lysis of some bacterial groups more than others. Therefore, some taxa will differ quite significantly in abundance depending on the method used (Figure 2).^72^ The primer selection will affect which variable region of the 16S rRNA gene is targeted, and this has a major impact on which taxa will be preferentially amplified and, therefore, reported in higher proportions.^72^ Most studies about gut microbiota target the variable region V4, but it has not been determined which one is preferable for dogs and cats.^73^ The choice of the sequencing platform, the chosen bioinformatics pipeline, and the reference database will also affect the reported proportions.^69^, ^74^

There are also major differences in how results are reported. Many authors report the analysis of bacterial taxa only on few phylogenetic levels, such as phylum or family, rather than all taxonomic levels. This can be misleading, as some taxonomic lineages can be highly diverse, as recently summarized by Lyu et al (see; https://www.frontiersin.org/files/Articles/556573/fmicb‐11‐01661‐HTML/image_m/fmicb‐11‐01661‐g001.jpg).^75^ For example, the phylum Firmicutes is highly diverse, consisting of various bacterial orders such as Clostridiales and Lactobacillales, with those again consisting of various genera and species. Some of these genera or species may be increased in intestinal disease (eg, *C perfringens*, *Streptococcus*), whereas others may be decreased (*C hiranonis*, *Faecalibacterium*).^49^, ^76^ Reporting results only on the phylum level could miss important information about these opposite changes on lower phylogenetic levels. Therefore, authors are encouraged to report, at least as supplemental information, their data with means and/or medians and ranges on all phylogenetic levels, even for taxa not significantly altered. There is also a high degree of identity of the 16S rRNA gene among genetically closely related genera, for example within Proteobacteria, like *Escherichia* and *Shigella*. This limits the use of the 16S rRNA gene for identifying opportunistic pathogenic bacteria on a species level, and more defined databases should be used for such purposes.^77^

Another limitation of current microbiome studies is that authors typically compare the effects of various environmental factors (eg, diet, collection and storage methods, breed influences, geographical location, etc) only to a control group or to its own baseline within the study, and in most cases with only a small sample size. Therefore, when changes are observed, it is difficult to extrapolate what the magnitude of observed changes are and how they compare against the wide range of normal microbiota in a large reference population or the targeted disease phenotype since there are no established RIs for bacterial taxa obtained using next‐generation sequencing. Also, no true analytical validation of 16S rRNA gene sequencing has been reported; and therefore, no information is available about the reproducibility of sequencing.

In summary, there is no single best approach for 16S rRNA gene sequencing of the microbiome, but understanding some of the limitations, choosing consistency in the methods, and using complementary techniques (NGS, quantitative PCR [qPCR], and metabolomics) across several studies might help to elucidate the underlying scientific questions. The identified taxa of interest can then be validated with a more reproducible method, such as qPCR.

## 5 QUANTITATIVE PCR—THE DYSBIOSIS INDEX

**FIGURE 3:**
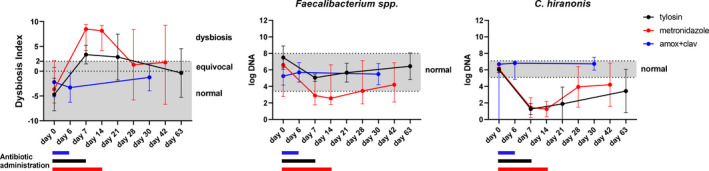
The effect of different antibiotics on canine fecal microbiota. The data are summarized from three different studies: dogs receiving tylosin (*n* = 8),^45^ metronidazole (*n* = 16),^43^ and amoxicillin‐clavulanic acid (*n* = 6).^84^ Dots indicate median values, error bars indicate ranges, grey areas indicate the RIs. All samples were analyzed using the same method (ie, DNA extraction and quantitative PCR assays),^49^ and this allows for a better comparison of data across different studies. Furthermore, the data can be compared with existing RIs, allowing conclusions to be drawn as to the magnitude of changes (size effect) of an intervention within the microbiota (Dysbiosis Index [DI]) or on specific bacterial taxa (ie, short‐chain fatty acid producing *Faecalibacterium* spp. and bile acid‐converting *C hiranonis*). These data show that broad‐spectrum antibiotics affect the abundance of *C hiranonis* (below RI), while amoxicillin‐clavulanic acid has a limited effect on the DI and *C hiranonis*

As mentioned above, 16S rRNA gene sequencing reports data as the relative abundance of bacterial taxa within a sample. Therefore, the changes in total bacterial load or abundance of specific taxa between samples are not assessed using an untargeted sequencing approach.^78^ For quantitation of total bacteria or individual taxa, qPCR is useful. It is a rapid (less than 24‐hour turnaround), affordable (lower equipment and per sample costs), and highly reproducible method to quantify specific taxa, which have been identified as clinically relevant based on previous sequencing studies.^49^, ^79^ Quantitative PCR has high reproducibility when the same methods are used (ie, DNA extraction, qPCR primers), and this allows the development of RIs for specific taxa. These reproducible assays can then be used to compare changes in bacterial abundance across studies and assess the magnitude of changes due to an intervention, as the results can be compared to an existing RI (Figure 3). The disadvantage of qPCR is that individual assays must be established for each target of interest.

An example of a qPCR approach is the canine microbiota DI.^49^ It measures the abundance of seven bacterial taxa and total bacteria and reports results individually for each bacterial group, as well as combines the abundances in a mathematical algorithm as a DI. These seven bacterial targets have been shown in several studies to be altered in dogs with CE^33^, ^34^, ^53^, ^80^ and antibiotic‐induced dysbiosis^42^, ^43^, ^45^, ^82^ using 16S rRNA gene sequencing. A DI cut‐off above 2 is currently considered dysbiosis, and the higher the DI, the more the microbiota diverges from normal.^29^, ^42^ Values between 0 and 2 are equivocal and indicate minor shifts in the microbiome. *Faecalibacterium*, *Fusobacterium*, *C hiranonis*, *Blautia*, and *Turicbacter* are typically decreased, and *Streptococcus* and *E coli* are increased in dysbiosis. The DI correlates with the results of 16S rRNA gene sequencing and correlates negatively with species richness (a higher DI indicates lower microbial diversity).^42^, ^43^, ^80^, ^83^ This assay allows for tracking how the microbiota changes after fecal microbiota transplantation or antibiotic usage.^42^, ^84^, ^85^ The DI predicts, by measuring the abundance of the bile acid 7alpha‐dehydroxylating bacterium, *C hiranonis*, the ability of the intestinal microbiota to convert primary to secondary bile acids. Several studies have shown that a high abundance of *C hiranonis* correlates with the presence of a higher percentage of secondary bile acids.^11^, ^15^, ^29^, ^44^, ^86^ The proper physiologic level of secondary bile acids in the intestine is important for the control of potential enteropathogens such as *C difficile*, *E coli*, and *C perfringens*.^40^, ^87^ Approx. 50%‐60% of dogs and 30% of cats with CE have a decreased abundance of *C hiranonis* and therefore decreased secondary BA.^48^, ^80^, ^88^ In humans, the germination of *C difficile* spores is promoted by a disrupted microbiota and consequently a reduction in secondary and increase in primary bile acids. Similarly, when dysbiosis is present in dogs, for example, due to intestinal inflammation or antibiotic use,^42^, ^43^, ^45^ this can lead to a lack of *C hiranonis*, lack of conversion from primary to secondary bile acids, and therefore the proliferation of *C difficile*. In an unpublished dataset from the author's laboratory, approx. 26% (315/1194) of dogs with chronic diarrhea tested positive for *C difficile*, and 80% of these lacked the bile acid converting bacterium, *C hiranonis*. Therefore, the overgrowth of *C difficile* might reflect the dysbiotic gut environment in CE, which has also been suggested in other studies.^89^, ^90^

## 6 IN‐SITU HYBRIDIZATION AND IMMUNOHISTOCHEMISTRY

To understand the role of bacteria in intestinal inflammation, it is useful to determine the localization of bacteria. This allows evaluating whether bacteria are within the mucus layer, attached to the epithelium, or located intracellularly. Bacteria can be visualized on biopsy slides either by using fluorescence in‐situ hybridization (FISH)^27^ or immunohistochemistry (IHC).^91^ FISH is the most commonly used approach, and probes are available that target all bacteria (eg, universal probes such as EUB338) and specific bacterial taxa.^21^, ^26^ The major advantage of FISH is that it enables one to visualize the localization of bacteria. Disadvantages are that specific probes need to be designed for bacteria of interest, and only a few probes can be used per tissue slide. This makes this approach labor‐intensive. Furthermore, expensive microscopy equipment is required, limiting FISH to few specialized laboratories.

While FISH is commonly performed on formalin‐fixed tissue, some data suggest that Carnoy's solution could be a better tissue fixative for FISH studies, as it better preserves the intestinal mucus layer, where bacteria of interest are located.^92^ This is especially the case for small intestinal biopsies in dogs and cats, as the thinner small intestinal mucus layer is often not well‐preserved in formalin‐fixed tissue slides, therefore, leading likely to an underestimation of attached bacteria.

Bacteria can also be enumerated in feces using FISH, and it has been reported that *Bifidobacteria* and *Bacteroides* decrease, and *Desulfovibrio* increase in cats with intestinal disease.^93^ One study using FISH on intestinal tissue reported that cats with inflammatory bowel disease (IBD) had increased numbers of Enterobacteriaceae adherent to the duodenal mucosa, and these correlated with changes in mucosal architecture.^10^ *E coli* and *Clostridium* spp. also correlated with intestinal inflammation suggesting that these bacteria contribute to the pathophysiology of IBD in cats. In another study, cats with small cell intestinal lymphoma had increased numbers of *Fusobacterium* sp. attached to the mucosa in the ileum and colon when compared with cats with IBD *Fusobacterium* sp. also correlated with increased expression of CD11b^+^ myeloid cells and NF‐κB.^21^ This association could suggest a potential contribution of bacteria to the development of small cell GI lymphoma in cats, as has been suggested in humans,^94^ but this requires identifying which species of *Fusobacterium* is involved and further mechanistic studies.

FISH has also been used to assess mucosa‐attached bacteria in healthy dogs and dogs with CE.^26^, ^27^, ^95^ Healthy dogs have an abundant microbiota (ie, *Helicobacter* spp.) in the colonic crypts (Figure 1), and these are depleted in dogs with CE.^27^ This suggests that bacteria in the colonic crypts provide beneficial immunologic properties. In contrast, the number of mucosa‐adherent bacteria is lower in healthy dogs and increased in dogs with CE.^27^

**FIGURE 4:**
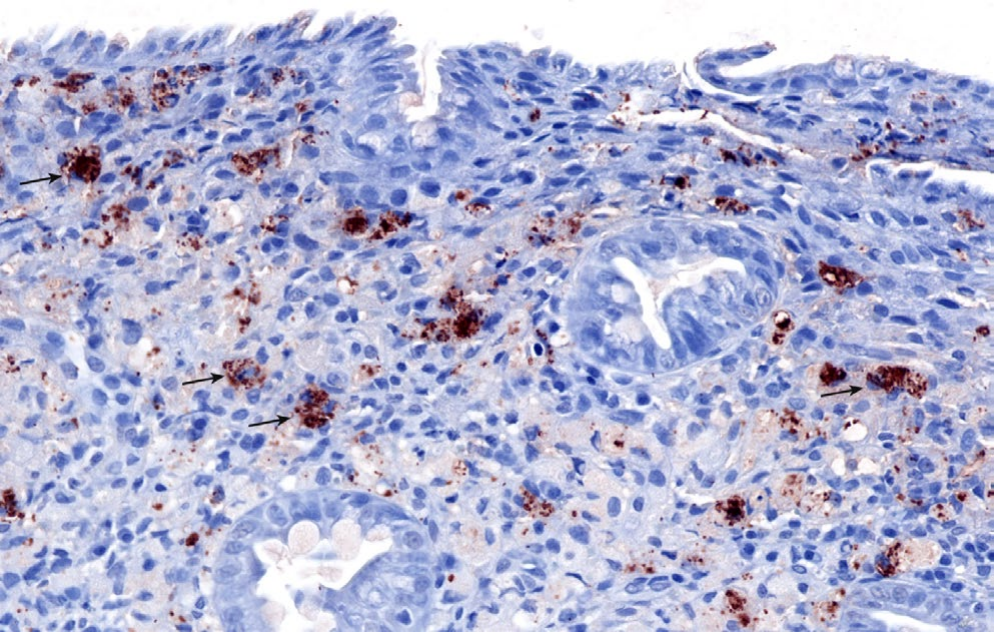
Photomicrograph of an intestinal biopsy from a dog with granulomatous colitis shows strong immunolabeling for *Escherichia coli* in the cytoplasm of macrophages in the lamina propria (arrows). Red diaminobenzidine chromogen and hematoxylin counterstain, ×20 objective. Courtesy of Dr Patricia Ishii, DVM, Texas A&M University and Dr Paula Giaretta, DACVP, Universidade Federal de Minas Gerais, Brazil

Generally, none or only a few intracellular bacteria are observed in dogs with CE. This is in contrast with granulomatous colitis associated with invasive *E coli*, most commonly observed in Boxer dogs and French Bulldogs.^96^, ^97^ These dogs respond well to antibiotic administration.^54^ For diagnosis, colonic biopsies can be stained using FISH, and the localization of bacteria within the intestinal tissue can be confirmed. However, as only few specialized laboratories perform FISH on a routine basis, it is often difficult for clinicians to submit samples and obtain results in a timely fashion. A recent case reported the successful identification of *E coli* using IHC in a dog with histiocytic ulcerative colitis.^91^ Therefore, IHC may be a more available option for the identification of intracellular *E coli* in the future (Figure 4).

## 7 METABOLOMICS

Metabolomics is an emerging and important area for the assessment of microbiota function and its contribution to health and disease. Microbial‐derived metabolites can be assessed either by targeted and validated assays that measure concentrations of already well‐understood microbial pathways (eg, SCFA, indoxyl‐sulfate, fecal bile acids) or by untargeted assays that measure several hundred different metabolites and are aimed for discovery. Most assays use mass spectrometry platforms.^98^ In the discovery phase, the measurement of metabolites should be combined with other phylogenetic assessment tools. Some functions of the microbiota, such as fermentation of dietary fiber to SCFA and the conversion of primary to secondary bile acids by some intestinal bacteria, are commonly studied. Several novel microbial pathways have been characterized in recent years, which affect gut, heart, and kidney function.

The dietary amino acid tryptophan is converted by intestinal bacteria into various indole metabolites. These play an important role in immunoregulation (eg, T‐cell response) within the intestine. Indole metabolites act as signaling molecules and can be anti‐inflammatory (eg, decrease IL‐8 expression), induce mucin gene expression, and strengthen tight junction resistance.^8^ Changes in the tryptophan‐indole pathways are associated with chronic enteropathy in dogs.^99^ Dietary supplementation with tryptophan has anti‐inflammatory effects in experimental colitis models and is likely a pathway of future investigation in dogs and cats.^100^

An increase in the serum concentration of trimethylamine N‐oxide (TMAO), a microbial‐derived product from the diet (ie, choline and L‐carnitine), is associated with atherosclerosis and cardiovascular disease in humans,^101^, ^102^ and with chronic heart failure in dogs.^13^, ^103^ Similarly, increased TMAO is associated with a poorer prognosis in CKD of people, likely due to its contribution to progressive renal tubulointerstitial fibrosis as shown in animal models.^101^ Other gut‐derived uremic metabolites, such as branched‐chain fatty acids, p‐cresol (microbial breakdown of tyrosine and phenylalanine), and indoxyl‐sulfate (from tryptophan), have also been associated with CKD in cats.^12^, ^104^ Future studies are warranted to understand which bacterial taxa are the main producer of these metabolites and whether dietary modulation (decrease of substrate) or direct microbiota modulation (eg, fiber, probiotics) might be used therapeutically.

## 8 CONCLUSIONS

Much progress has been made over the last several years to better define the intestinal microbiota and their metabolic and immunoregulatory contributions to health and disease. Various complementary tools that assess the microbiota and metabolic pathways are available. In understanding their limitations as well as advantages, specific approaches can be applied to pathway discovery or defined research studies. Initial assays and RIs have been established for specific clinical applications (eg, dysbiosis index, FISH for *E coli*). As for other organ systems, it is very likely that with time more metabolic pathways and bacterial taxa will be identified as additional microbial biomarkers.

## DISCLOSURE

The author is an employee of the Gastrointestinal Laboratory at Texas A&M University that offers microbiome and gastrointestinal function testing on a fee‐for‐service basis.
